# Molecular characterization and immunoprotective potential of microneme protein 3 from *Eimeria necatrix*

**DOI:** 10.1186/s13071-026-07418-w

**Published:** 2026-05-02

**Authors:** Qianqian Feng, Danli Yan, Nianyu Xue, Dandan Liu, Weimin Cai, Yuxin Zhou, Zhaofeng Hou, Jinjun Xu, Jianping Tao

**Affiliations:** 1 College of Veterinary Medicine, Yangzhou University, 48 East Wenhui Road, Yangzhou, Jiangsu 225009 People’s Republic of China; 2 Jiangsu Co-innovation Center for Prevention and Control of Important Animal Infectious Diseases and Zoonoses, Yangzhou University, Yangzhou, 225009 People’s Republic of China; 3 College of Bioscience and Biotechnology, Yangzhou University, Yangzhou, 225009 People’s Republic of China

**Keywords:** *Eimeria necatrix*, MIC3, EGF-like domain, Invasion, Protective efficacy

## Abstract

**Background:**

*Eimeria necatrix*, a member of the Apicomplexa phylum, is one of the most pathogenic parasites, causing high mortality in chickens. Microneme proteins (MICs) play essential roles in host cell recognition and invasion by apicomplexan parasites and are also attractive candidates for vaccine development. However, comprehensive studies on *E. necatrix* MICs remain limited.

**Methods:**

*Eimeria necatrix MIC3* gene (En*MIC3*) was amplified and expressed in *Escherichia coli*. The recombinant protein (rEnMIC3) was characterized via SDS-PAGE and Western blot. The antigenicity of rEnMIC3 and its localization in sporozoites (SZ) and second-generation merozoites (MZ-2) of *E. necatrix* were determined by Western blot and indirect immunofluorescence analyses (IFAs). The dynamic expression of EnMIC3 across different developmental stages and its impact on sporozoite invasion of host cells were analyzed. The immune protection provided by rEnMIC3 was evaluated in chickens using weight gain, lesion scores, oocyst production, anticoccidial index (ACI), and antibody levels.

**Results:**

The open reading frame of En*MIC3* was 798 bp, encoding a 265-amino acid protein with a predicted molecular weight of 28.50 kDa. EnMIC3 contained a signal peptide and a single epidermal growth factor (EGF)-like domain. The rEnMIC3 with an approximate molecular weight of 36 kDa could be specifically recognized by convalescent sera from chickens infected with *E. necatrix*. The molecular mass of the native protein was approximately 35 kDa, and it localizes to the apical region in SZ but exhibits a cytoplasmic distribution in MZ-2. EnMIC3 mRNA was expressed at significantly higher levels in SZ than in MZ-2, whereas protein expression displayed an inverse pattern. Anti-rEnMIC3 polyclonal antibodies inhibited sporozoite invasion of DF-1 cells in a dose-dependent manner. Vaccination with rEnMIC3 conferred effective protection against *E. necatrix* challenge, with the high-dose group (200 µg) achieving the highest ACI value (171.32) and markedly elevated serum antibody levels.

**Conclusions:**

These findings not only offer a foundation for understanding the role of EnMIC3 protein in the host invasion of *E. necatrix* but also present a potential protective antigen of *E. necatrix* for the development of a subunit vaccine against avian coccidiosis.

**Graphical Abstract:**

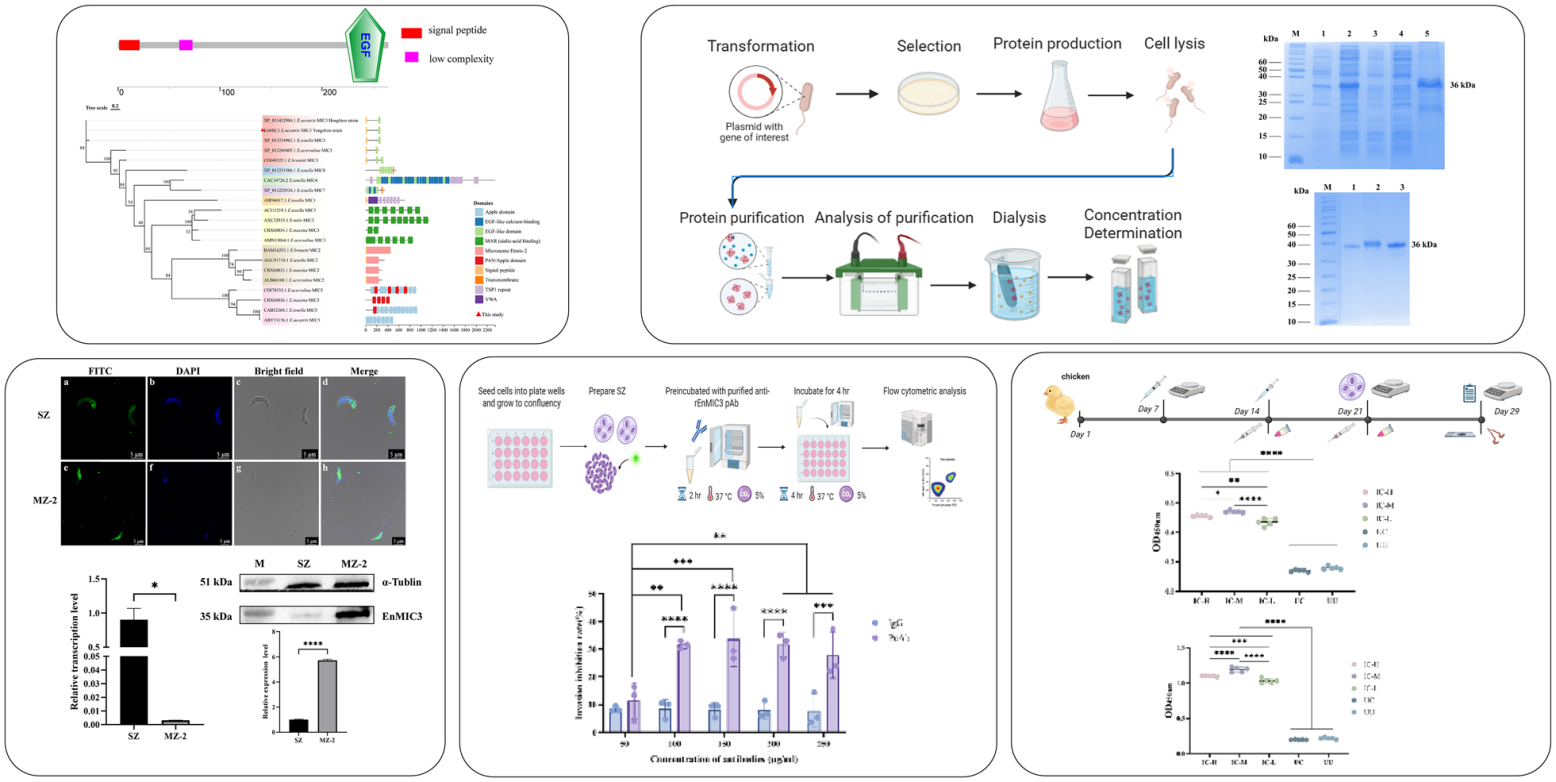

**Supplementary Information:**

The online version contains supplementary material available at 10.1186/s13071-026-07418-w.

## Background

Chicken coccidiosis is a parasitic protozoan disease caused by the genus *Eimeria*, which exhibits strict host specificity and causes significant economic losses to the global poultry industry annually. According to a 2023 survey, coccidiosis ranks as the most prevalent disease in the broiler poultry industry [1]. Traditionally, seven *Eimeria* species were recognized in chickens; however, three additional species, *Eimeria lata, E. nagambie,* and *E. zaria*, have been described more recently [2]. Among these species, *Eimeria necatrix* is considered one of the most pathogenic, primarily colonizing the middle portion of the small intestine and causing severe hemorrhagic enteritis that can lead to high mortality rates in infected flocks. In recent years, the prevalence of *E. necatrix* has shown an increasing trend in China, with a detection rate of 50.95% in fecal samples from 634 poultry farms across 15 provinces, which was lower than that of *Eimeria acervulina* (65.62%) but higher than that of *Eimeria tenella* (48.42%) [3]. Current control of coccidiosis is achieved through good husbandry practices, anticoccidial drugs, and/or live parasite vaccination [1]. Unfortunately, long-term drug use has inevitably led to the emergence of drug resistance, as well as the accumulation of drug residue in the food chain and environment [4, 5]. Live vaccines, both non-attenuated and attenuated, have shown effectiveness against coccidiosis [6]. However, the commercial use of live anticoccidial vaccines is limited by the risk of vaccine-induced pathogenicity, antigenic diversity, high production costs, and limited production capacity [7, 8]. Recent studies have demonstrated that recombinant proteins can effectively stimulate immune responses in chickens to resist *Eimeria* infection [9, 10]. These findings highlight the urgent need to develop novel, safe, cost-effective recombinant subunit vaccines against avian coccidiosis.

Apicomplexan parasites are obligate intracellular pathogens whose ability to invade and egress from host cells is essential for their survival and dissemination. Successful invasion of host cells represents a critical determinant for establishing infection [11]. Microneme proteins (MICs), secreted from specialized apical organelles called micronemes, play pivotal roles in host cell recognition, attachment, and invasion. These proteins are sequentially released onto the parasite surface during the invasion process and mediate essential interactions with host cell receptors [12–14]. Owing to their important roles in the early stages of infection and their exposure to the host immune system, MICs are regarded as promising targets for vaccine development[15]. In *Eimeria*, several MIC proteins have been identified and shown to contribute to parasite invasion and development within host cells. In addition, several recombinant MIC proteins, such as *Eimeria tenella* MIC1–3,* E. acervulina* MIC3, and *Eimeria mitis* MIC3, have demonstrated partial protective efficacy in immunization studies, supporting their potential value as anticoccidial vaccine candidates [16–22]. However, comprehensive studies on *E. necatrix* MICs remain limited.

In our previous proteomic analysis of unsporulated oocysts, sporozoites, and second-generation merozoites of *E. necatrix*, six EnMIC proteins were identified [23, 24]. In the present study, we cloned and expressed the microneme 3 gene from *E. necatrix* (EnMIC3) and analyzed its molecular characterization, dynamic expression across different developmental stages, and in vivo localization. Additionally, we evaluated the impact of EnMIC3 on sporozoite invasion of host cells and its protective efficacy for the first time to our knowledge. Our results provide a candidate antigen for controlling this parasite and lay a foundation for further exploration of the molecular mechanism underlying sporozoite invasion mediated by EnMIC3.

## Methods

### Parasites and animals

The oocysts of *E. necatrix* Yangzhou strain used in this study were maintained in our laboratory. The identity of the strain was determined using the single oocyst method, followed by microscopic examination and sequencing of the internal transcribed spacer (ITS) region of the ribosomal RNA (rRNA) gene [25]. Propagation, harvesting, and sporulation of oocysts were performed as previously described [26].

One-day-old, yellow-feathered broilers were purchased from Jiangsu Lihua Animal Husbandry Co., Ltd. (Changzhou, Jiangsu, China) and raised under coccidia-free conditions. Anticoccidial-free feed and water were provided ad libitum. Female BALB/c mice aged 6 to 8 weeks were purchased from the Experimental Animal Center of Yangzhou University (Yangzhou, Jiangsu, China).

All animal procedures were approved by the Animal Experiment Ethics Committee of Yangzhou University and conducted in accordance with its guidelines and regulations [license no. SYXK (SU) 2021-0027].

### Preparation of different developmental stages of* E. necatrix*

Sporulated oocysts were obtained by incubating unsporulated oocysts in 2.5% potassium dichromate solution at room temperature for 2–3 days. Sporozoites (SZ) were prepared as previously described [24]. Briefly, freshly sporulated oocysts were sterilized in 20% sodium hypochlorite for 5–10 min at 4 °C and washed three times with phosphate-buffered saline (PBS) by centrifugation at 1,400 × *g* for 5 min. The oocysts were then ground with glass beads (150–212 µm in diameter; Sigma-Aldrich, St. Louis, MO, USA) to release sporocysts. Sporozoites were excysted by incubation in sterile PBS containing 0.75% (w/v) trypsin (Solarbio, Beijing, China) and 10% (w/v) bile at 41 °C for 40–60 min. Free sporozoites were collected by filtration through a G3 funnel and stored in liquid nitrogen until use.

The second-generation merozoites (MZ-2) were isolated from the intestines of chickens orally infected with 1.0 × 10^4^ sporulated *E*. *necatrix* oocysts at 138 h post-infection. The mucosa was scraped using a glass slide, and the collected scrapings were homogenized and incubated in digestion buffer (120 mM NaCl, 10 mM CaCl_2_, 3 mM K_2_HPO_4_, 20 mM Tris-HCl, 0.1% BSA, 0.1% hyaluronidase) at 37 °C for 1 h. The crude merozoite suspension was filtered through gauze, followed by sequential filtration through 17- and 10-µm polyamide meshes (Sefar, Suzhou, China). The filtrate was then treated with erythrocyte lysis buffer (Solarbio, Beijing, China) to lyse chicken erythrocytes. Merozoites were resuspended in 30% Percoll in PBS (P-PBS), and five volumes of the suspension were gently layered onto one volume of 50% P-PBS and centrifuged at 2,200 × *g* for 15 min. The pellet containing merozoites was collected and stored at − 80 °C until use.

### Cloning, sequencing, and bioinformatic analysis of EnMIC3

Total RNA was extracted from the SZ of *E. necatrix* using the RNA Extraction Kit™ (TaKaRa, Tokyo, Japan) in accordance with the manufacturer’s protocol. The RNA was treated with DNase I and reverse transcribed into cDNA using reverse transcriptase (TaKaRa, Dalian, China) and oligo(dT) primers. The full-length *EnMIC3* gene (GenBank accession no. XM_013578447.1) was amplified from cDNA using the primers En*MIC3*-F and En*MIC3*-R (Table 1).

**Table 1:** List of primer sequences

Name of primer	Sequence (5' to 3')
En*MIC3*-F^a^	ATGTATTCGAAAATACTGCTGGTGG
En*MIC3*-R^a^	CTAGTAGTTGAACACGCACTG
En*MIC3*-pET28aF^b^	ATGGGTCGCGGATCCGAATTCTCTGCGCAGCGGGCCACG
En*MIC3*-pET28aR^b^	GTGGTGGTGGTGGTGCTCGAGCTAGTAGTTGAACACGCACTGTTCC
qEn*MIC3*-F	GCGGGCGTTTCGGACTTTC
qEn*MIC3*-R	GGTTGAACTCCTCTTCGGTGATG
5.8S rRNA-F	TTCATACTGCGTCTAATGCACC
5.8S rRNA-R	CGAGTCCCTACCGCAGTACTA

^a^Primers for complete gene

^b^Primers for prokaryotic expression

A bioinformatic analysis of EnMIC3 was performed using multiple online tools. The physicochemical properties, including molecular weight, amino acid composition, and theoretical isoelectric point (pI), were analyzed using ExPASy ProtParam (https://web.expasy.org/protparam/). Signal peptides were predicted using SignalP 5.0 (https://services.healthtech.dtu.dk/services/SignalP-5.0/), and transmembrane domains were predicted using TMHMM 2.0 (https://services.healthtech.dtu.dk/services/TMHMM-2.0/). Functional domains were identified using the SMART database (http://smart.embl-heidelberg.de/). To investigate the evolutionary relationships of EnMIC3 with other microneme proteins (MICs) across *Eimeria* species, amino acid sequences of previously characterized MICs (MIC1–MIC5, MIC7, and MIC8) from *Eimeria necatrix*, *E. tenella*, *E. acervulina*, *E. maxima*, *E. brunetti*, and *E. mitis* were retrieved from the NCBI database (https://www.ncbi.nlm.nih.gov/). Multiple sequence alignment was performed using MAFFT v7 (https://mafft.cbrc.jp/alignment/server/) with default parameters. A maximum likelihood (ML) phylogenetic tree was constructed using IQ-TREE 2 [27], with 1000 replicates for branch support assessed by Approximate Likelihood Ratio Test (aLRT) and ultrafast bootstraps, respectively. The conserved functional domains of each protein were predicted using the SMART database integrated in InterPro (https://www.ebi.ac.uk/interpro/). The phylogenetic tree and domain architecture were integrated and visualized using ChiPlot (https://www.chiplot.online/) [28].

### Expression and purification of recombinant protein

To construct the expression vector, a gene fragment encoding the mature EnMIC3 protein (aa 20-265, excluding the signal peptide) was PCR-amplified from the pGEM®-T Easy vector using specific primers with restriction sites at their 5′ ends (Table 1). The PCR products were gel-purified and subsequently ligated into the pET28a(+) vector at the *EcoR* I and *Xho* I sites. The recombinant vector (designated pET28a(+)-EnMIC3) was verified by restriction endonuclease digestion and DNA sequencing (Beijing Genomics Institution, Beijing, China).

Both the empty pET28a(+) vector and pET28a(+)-EnMIC3 construct were transformed into *Escherichia coli* Transetta (DE3) (TransGen Biotech, Beijing, China). Following induction with isopropyl-β-D-thiogalactopyranoside (IPTG; Solarbio, Beijing, China), the rEnMIC3 was expressed predominantly as inclusion bodies and purified using Ni Sepharose™ Excel resin (GE Healthcare, Piscataway, NJ, USA). The inclusion bodies were solubilized in 8 M urea, and purified recombinant proteins were refolded by renaturation buffer (50 mM Tris–HCl, 0.15 M NaCl, pH 8.0) containing different urea concentrations (6, 4, 2, 1, 0 mol/l). The refolded protein was then concentrated using polyethylene glycol (PEG 8000). The purity of protein samples was assessed via sodium dodecyl sulfate-polyacrylamide gel electrophoresis (SDS-PAGE) analysis.

### Antibody preparations

Polyclonal antibodies against rEnMIC3 were generated following a previously described method [25]. Briefly, 20 µg of purified protein was resuspended in 50 µl of sterile PBS and mixed with 50 µl of Quick Antibody-Mouse 3W adjuvant (Biodragon, Beijing, China). Six-week-old BALB/c mice were immunized twice according to the manufacturer's recommendations. Eight days after the final immunization, sera were collected from the immunized mice and tested for reactivity. Convalescent chicken sera, prepared from chickens individually infected with *E. necatrix*, *E. tenella*, *E. acervuline*, or *E. maxima*, had been previously prepared and stored at -80 ℃ in our laboratory.

### Western blot analysis

The purified rEnMIC3 was first resolved by SDS-PAGE and then transferred onto nitrocellulose membranes (Merck Millipore, Billerica, MA, USA). After blocking with 5% bovine serum albumin (BSA) in Tris-buffered saline containing 0.05% Tween-20 (TBST), the membranes were incubated with primary antibodies, followed by incubation with the corresponding horseradish peroxidase (HRP)-conjugated secondary antibodies. The membranes were washed five times with TBST for 5 min each and visualized using a Hypersensitive ECL Chemiluminescence Kit (Tanon, Shanghai, China) for 1 min in a dark room. Membrane imaging was performed using the Tanon-5200 Chemiluminescent Imaging System (Tanon, Shanghai, China).

For protein extraction, purified SZ and MZ-2 were lysed in radioimmunoprecipitation assay (RIPA) buffer containing a protease inhibitor cocktail (Beyotime Biotechnology, Shanghai, China) on ice for 1 h. The lysates were centrifuged at 10,000 × g for 30 min at 4 °C, and the supernatants were collected. Total protein concentrations were determined using an Enhanced BCA Protein Assay Kit (Beyotime Biotechnology, Shanghai, China) before loading, and equal amounts of protein were mixed with denaturing sample buffer (50 mM Tris–HCl [pH 6.8], 2% SDS, 0.1% bromophenol blue, 10% glycerol, and 100 mM DTT), boiled, separated by SDS-PAGE, and transferred onto nitrocellulose membranes for Western blot analysis. Membranes were probed with anti-rEnMIC3 pAb (1:200 dilution), and α-tubulin was used as the loading control. Band intensities were quantified by densitometric analysis and normalized to tubulin.

### Immunofluorescence analysis

IFA was used to localize EnMIC3 and was performed as previously described [25]. Briefly, fresh SZ and MZ-2 were fixed, permeabilized, and blocked. The samples were incubated with an anti-rEnMIC3 pAb (1:100 dilution) as the primary antibody and FITC-conjugated goat anti-mouse IgG (1:100 dilution; KPL, Gaithersburg, MD, USA) as the secondary antibody. Nuclei were counterstained using DAPI-containing antifade mounting medium (Roche, Basel, Switzerland). Fluorescent images were acquired using a laser scanning confocal microscope (Leica TCS SP8 STED, Wetzlar, Germany).

### Transcript levels of EnMIC3 in different developmental stages of *E. necatrix*

Total RNA from SZ and MZ-2 was extracted using an RNA Extraction Kit™ (TaKaRa, Tokyo, Japan) following the manufacturer’s protocol. The cDNA was synthesized using the PrimeScript RT Reagent Kit with gDNA Eraser (TaKaRa., Shiga, Japan). Specific primers (Table 1) were designed to amplify EnMIC3 cDNA by quantitative PCR (qPCR). *Eimeria necatrix* 5.8S ribosomal RNA (5.8S rRNA) was used as an internal control. qPCR was performed with the AceQ® qPCR SYBR Green Master Mix (Vazyme, Nanjing, China) following the manufacturer’s protocol. Each sample was analyzed in biological triplicate, and three independent experiments were performed. The relative expression of EnMIC3 mRNA was calculated using the 2^−ΔΔCt^ method [29]. Differences were considered statistically significant at *P* < 0.05.

### Invasion inhibition assay

The invasion inhibition assay was performed based on the ability of *E. necatrix* sporozoites to invade DF-1 cells, as described previously [10]. DF-1 cells were seeded at a density of 1 × 10^5^ cells per well in 24-well plates and cultured in DMEM (Gibco, Grand Island, NY, USA) supplemented with 10% fetal bovine serum (FBS; Lonsera, Shanghai, China) and 500 U/ml penicillin/streptomycin (Beyotime Biotechnology, Shanghai, China) for 8 h at 37 °C and 5% CO_2_. Freshly harvested SZs were labeled with CFSE (Beyotime Biotechnology, Shanghai, China) according to the manufacturer’s instructions. CFSE-labeled SZs (6.0 × 10^5^/well) were then preincubated with purified anti-rEnMIC3 pAb (Additional file 1: Fig. S1) at concentrations of 50, 100, 150, 200, and 250 µg/ml in complete medium for 2 h at 37 °C. Following preincubation, the SZs were added to DF-1 cell monolayers and incubated for 4 h at 41 °C in 5% CO₂ to allow invasion. After incubation, the infected monolayers were washed with PBS, digested with 0.25% trypsin (Gibco, Grand Island, NY, USA), and collected for flow cytometric analysis using a CytoFLEX flow cytometer (Beckman Coulter, USA). The invasion inhibition rate was calculated based on the percentage of uninvaded sporozoites. Each treatment was performed in triplicate. Mouse IgG (Biodragon, Beijing, China) at equivalent concentrations served as the negative control, while untreated CFSE-labeled sporozoites served as the blank control.

### Evaluation of immune protection

The experimental design and immunization procedures were based on published studies [10, 30] and are summarized in Table 2. One hundred 7-day-old chickens with similar body weight were randomly divided into five groups (*n* = 20, for each): three immunized-challenged (IC) groups, including a low-dose group (50 µg, IC-L), a medium-dose group (100 µg, IC-M), and a high-dose group (200 µg, IC-H), as well as two control groups consisting of an unimmunized challenged group (UC) and an unimmunized unchallenged group (UU).

**Table 2:** Experimental design and immunization program

Groups	No. of chickens	Immunization dose (μg)	Immunization (d)^*^	Immunization route	Challenge (d)	Challenge dose (× 10^4^ oocysts)
IC-H	20	200	7, 14	S.C.^**^	21	2.5
IC-M	20	100	7, 14	S.C.	21	2.5
IC-L	20	50	7, 14	S.C.	21	2.5
UC	20	PBS	7, 14	S.C.	21	2.5
UU	20	PBS	7, 14	S.C.	/	/

^*^On day 7, rEnMIC3 protein emulsified with Freund’s complete adjuvant (FCA; Sigma) at a 1:1 (v/v) ratio; on day 14, rEnMIC3 protein emulsified with Freund’s incomplete adjuvant (FIA; Sigma) at a 1:1 (v/v) ratio

^**^Subcutaneous injection route

Chickens in the IC-H, IC-M, and IC-L groups were immunized subcutaneously in the thigh with 200, 100, and 50 µg rEnMIC3, respectively, in a total volume of 0.2 ml per bird. For the primary immunization on day 7, rEnMIC3 was emulsified with Freund’s complete adjuvant (FCA; Sigma) at a 1:1 (v/v) ratio. For the booster immunization on day 14, the same dose of rEnMIC3 was emulsified with Freund’s incomplete adjuvant (FIA; Sigma) at a1:1 (v/v) ratio. Chickens in the UC and UU groups received the same volume of PBS alone via the same route. On day 21, chickens in the IC groups and the UC group were orally challenged with 2.5 × 10^4^ sporulated oocysts of *E. necatrix* Yangzhou strain. On day 29 (8 days post-challenge), the chickens were killed, and the small intestines were collected for lesion examination.

The protective efficacy was evaluated based on survival rate (SR), body weight gain (BWG), oocyst reduction (OR), lesion score (LS), and anticoccidial index (ACI). The survival rate was calculated as the proportion of surviving chickens relative to the total number of chickens in each group. BWG was determined by calculating the difference in body weight between day 21 (post-booster immunization) and day 29 (8 days post-infection). The feces of each group were collected every 12 h on days 6 to 8 post-infection, and the cecal contents of each group were collected at the end of the test. The oocysts in feces and cecal contents were determined using McMaster’s counting technique. The total oocyst production (OP) per bird from feces and ceca was used to calculate OR using the formula: ((oocyst output of UC group − oocyst output of IC groups)/oocyst output of UC group) × 100%. The intestinal lesions were scored on a scale of 0 to 4, using the method described by Johnson and Reid [30].

ACI value is calculated using the formula: (SR + RBWG (%) − (LI (lesion index) + OI (oocyst index)) according to the previous description [31]. RBWG (%) is calculated using the formula: (BWG of IC or UC groups/BWG of UU group) × 100%. LI is calculated by multiplying the average LS by 10. OI is based on the ratio of oocyst output of IC groups to that of UC group. This ratio is divided into five grades: 0–1%, 1–25%, 26–50%, 51–75%, and 76–100%, with corresponding OI values recorded as 0, 5, 10, 20, and 40, respectively. An ACI value of ≥ 180 was considered high performance, an ACI value between 160 and 179 was considered effective, and an ACI value of < 160 was deemed ineffective [31].

Serum antibody levels were measured by enzyme-linked immunosorbent assay (ELISA) as described previously [10]. Blood samples were collected from five randomly selected chickens per group on day 14 (prior to booster immunization) and on day 21 (prior to challenge). All samples were analyzed in triplicate.

### Statistical analysis

Data are presented as mean ± SD. Statistical analyses and graphs were generated using GraphPad Prism software (GraphPad version 10.1.2, CA, USA). Normality was assessed using the Shapiro-Wilk test prior to statistical analysis. For normally distributed data, comparisons between two groups were performed using Student’s t-test, whereas comparisons among three or more groups were analyzed using one-way ANOVA followed by Tukey’s multiple comparisons test. For non-normally distributed data, the Kruskal-Wallis test followed by Dunn’s multiple comparisons test was used. Significance levels are indicated as *P < 0.05, **P < 0.01, ***P < 0.001, and ****P < 0.0001.

## Results

### Identification of *E. necatrix* MIC3

**Fig. 1:**
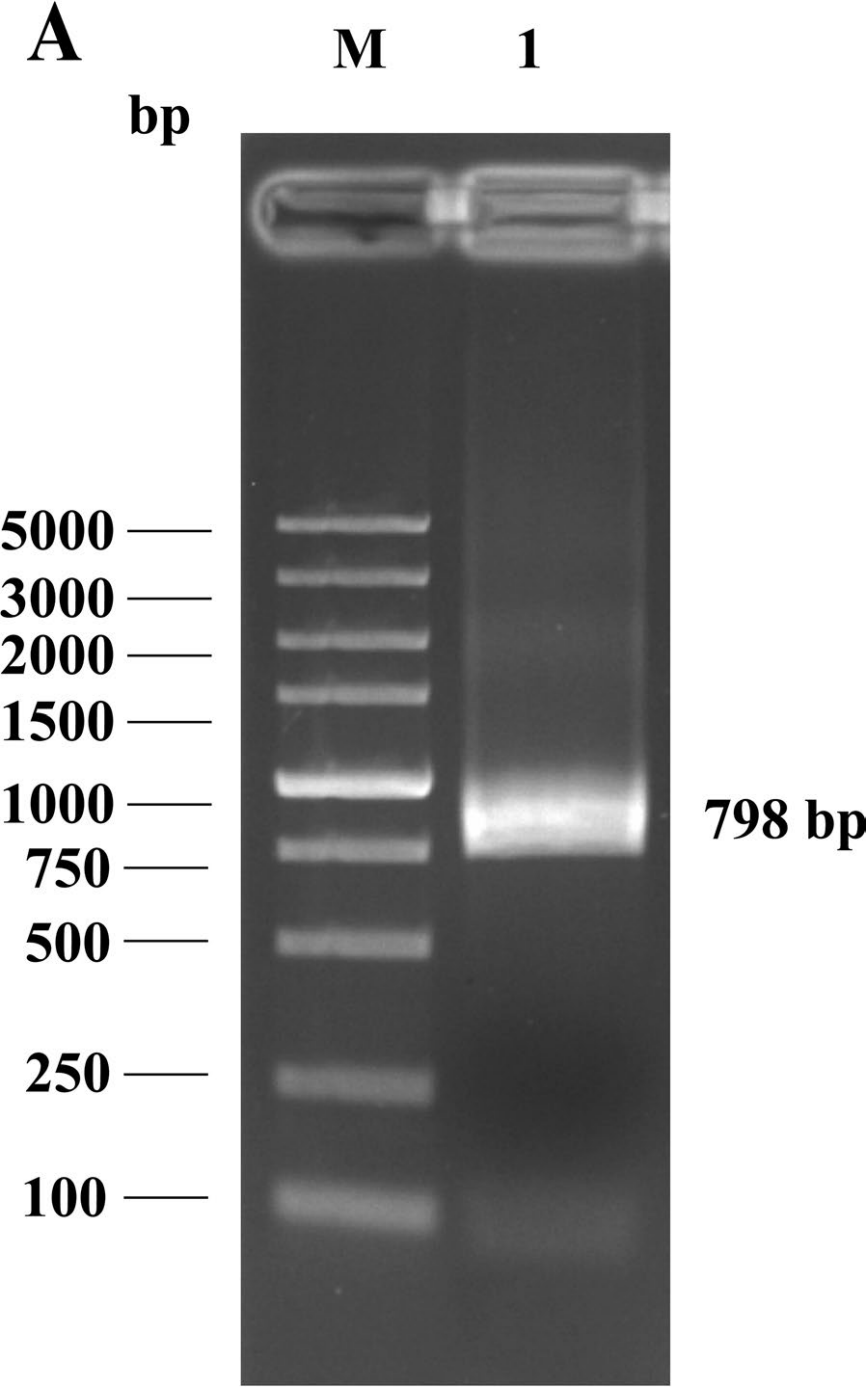
RT-PCR amplification of the En*MIC3* gene from *Eimeria necatrix*. M: DL5000 DNA Marker; Lane 1: En*MIC3* RT-PCR product

**Fig. 2:**
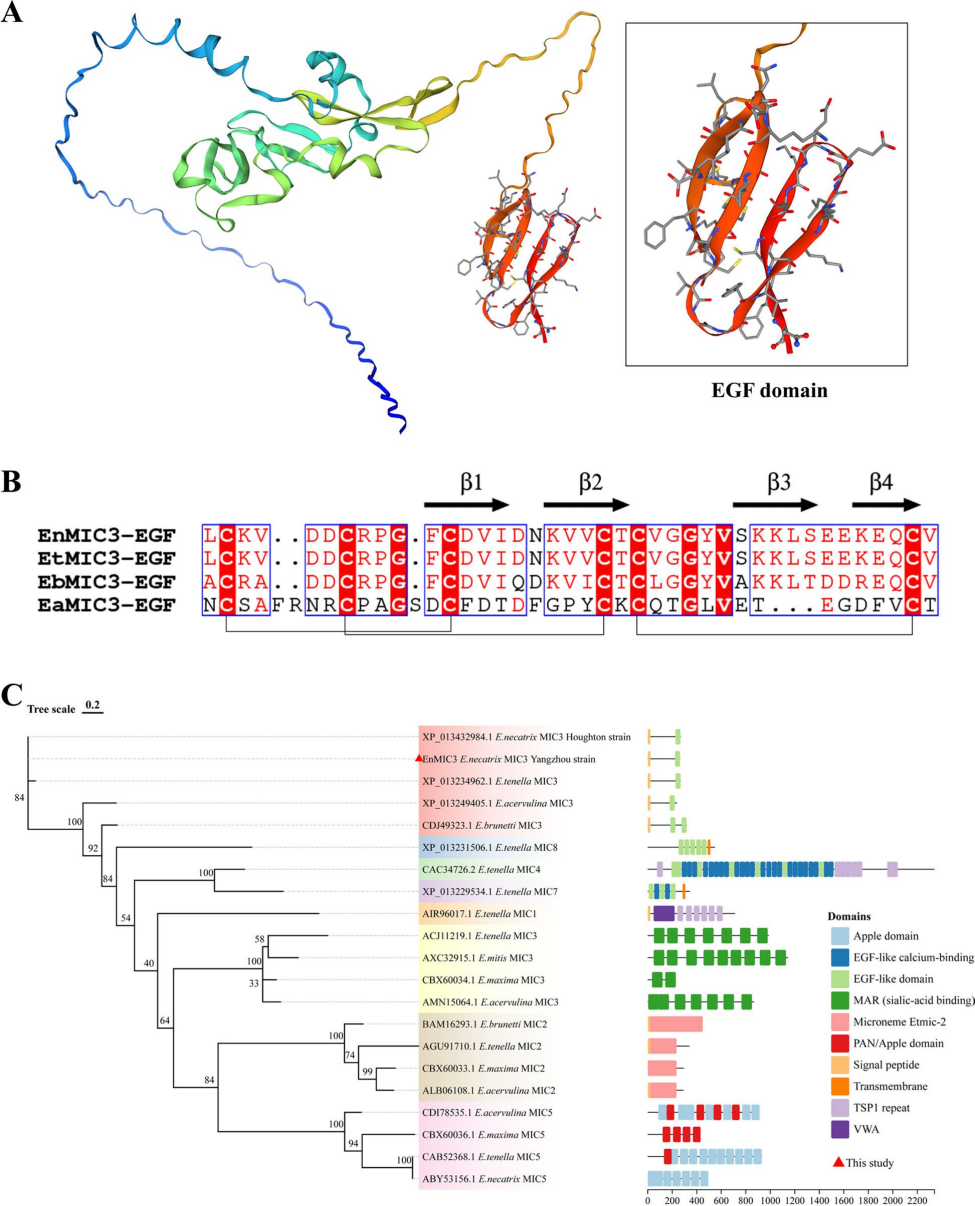
Identification and characterization of EnMIC3. **A** Predicted tertiary structure of EnMIC3 protein. The EGF domain is highlighted in the boxed region. **B** Sequence alignment of the EGF domains from four *Eimeria* species (EnMIC3, EtMIC3, EbMIC3, and EaMIC3). The six conserved cysteine residues are indicated by boxes, and the predicted β-sheets (β1–β4) are shown above the alignment. **C** Maximum likelihood phylogenetic tree was constructed using IQ-TREE 2, with 1000 replicates for branch support assessed by approximate likelihood ratio test (aLRT) and ultrafast bootstrap, respectively. Conserved functional domains were predicted using the SMART database integrated in InterPro, with different colors representing distinct domain types as indicated in the legend. The red triangle (▲) indicates the EnMIC3 identified in this study. NCBI accession numbers were employed

An En*MIC3* cDNA was cloned from the *E. necatrix* Yangzhou strain, with a full length of 798 bp (Fig. 1). Homology analysis revealed 99.7% identity to the published GenBank sequence (XM_013577530.1), with a 12-bp deletion located between − 651 bp and − 662 bp upstream of the start codon (Additional file 1: Fig. S2). The open reading frame (ORF) of the EnMIC3 cDNA encodes a protein consisting of 265 amino acids, with a predicted molecular mass of 28.50 kDa and a theoretical isoelectric point (pI) of 5.23. Bioinformatic analysis revealed that the protein lacks transmembrane domains and possesses seven predicted antigenic epitopes. The predicted protein structure comprises an N–terminal signal peptide (aa 1–19), a low-complexity region (aa 60–73), and an epidermal growth factor (EGF)-like domain (aa 223–262) but lacks a microneme adhesive repeat (MAR) domain. The tertiary structure of EnMIC3 was predicted using SWISS-MODEL, and the resulting structural model revealed a characteristic EGF domain within its conserved region (Fig. 2A). Homology searches using the amino acid sequence of EnMIC3 identified three orthologous genes in chicken *Eimeria* species. Pairwise sequence comparisons showed that EnMIC3 shared the highest identity (92.5%) with *E. tenella* MIC3 (XP_013234962.1), followed by 52.8% identity with *E. acervulina* MIC3 (XP_013249405.1) and 47.3% identity with *E. brunetti* MIC3 (CDJ49323.1) (Additional file 1: Fig. S3). To further characterize the EGF domain across *Eimeria* species, multiple sequence alignment was performed using the EGF domain sequences from EnMIC3, EtMIC3, EbMIC3, and EaMIC3 (Fig. 2B). The alignment revealed that six cysteine residues were strictly conserved among all four species, representing a hallmark feature of the EGF domain. These conserved cysteines are predicted to form three intramolecular disulfide bonds essential for maintaining the domain's structural integrity. Additionally, secondary-structure prediction identified four β-strands (β1–β4) in this region, consistent with the canonical EGF fold architecture.

Phylogenetic analysis revealed that the *Eimeria* MIC proteins clustered into distinct clades corresponding to different MIC families (Fig. 2C). EnMIC3 from the *E. necatrix* Yangzhou strain (this study) grouped closely with the MIC3 ortholog from the *E. necatrix* Houghton strain (XP_013432984.1) and formed a well-supported clade with MIC3 orthologs from *E. tenella*, *E. acervulina*, and *E. brunetti*. These short-form MIC3 proteins shared a conserved, relatively simple domain architecture consisting of a signal peptide and one or two EGF-like domains. This clade was sister to *E. tenella* MIC8, which possessed a distinct domain organization containing five EGF-like domains and a transmembrane domain. *Eimeria tenella* MIC4 and MIC7 clustered together as a neighboring clade, both harboring multiple EGF-like and EGF-like calcium-binding domains, with MIC4 exhibiting the largest protein size among all analyzed MICs (> 2000 amino acids). The *E. tenella* MIC1 (AIR96017.1) occupied an independent phylogenetic position, branching separately from the above EGF-domain-rich clades. In contrast to those families, MIC1 had a distinct domain architecture comprising a von Willebrand factor A (VWA) domain followed by multiple thrombospondin type I (TSP-1) repeats. Notably, a separate group of long-form MIC3 proteins from *E. tenella* (ACJ11219.1), *E. mitis* (AXC32915.1), *E. maxima* (CBX60034.1), and *E. acervulina* (AMN15064.1) formed an independent clade distinct from the short-form MIC3 group, characterized by a substantially greater number of MAR domains. This pattern suggests that the MIC3 gene family has undergone lineage-specific domain expansion events during *Eimeria* evolution, giving rise to two structurally distinct forms. The MIC2 proteins from *E. brunetti*, *E. tenella*, *E. maxima*, and *E. acervulina* formed a well-supported clade, characterized by a Microneme Etmic-2 domain, while the MIC5 orthologs from *E. acervulina*, *E. maxima*, *E. tenella*, and *E. necatrix* clustered together, sharing a conserved architecture comprising PAN/Apple and Apple domains. Taken together, these results confirmed that the EnMIC3 identified in this study is a typical short-form MIC3 ortholog, phylogenetically and structurally distinct from other MIC families and long-form MIC3 proteins.

### Expression, purification, and immunological characterization of rEnMIC3

**Fig. 3:**
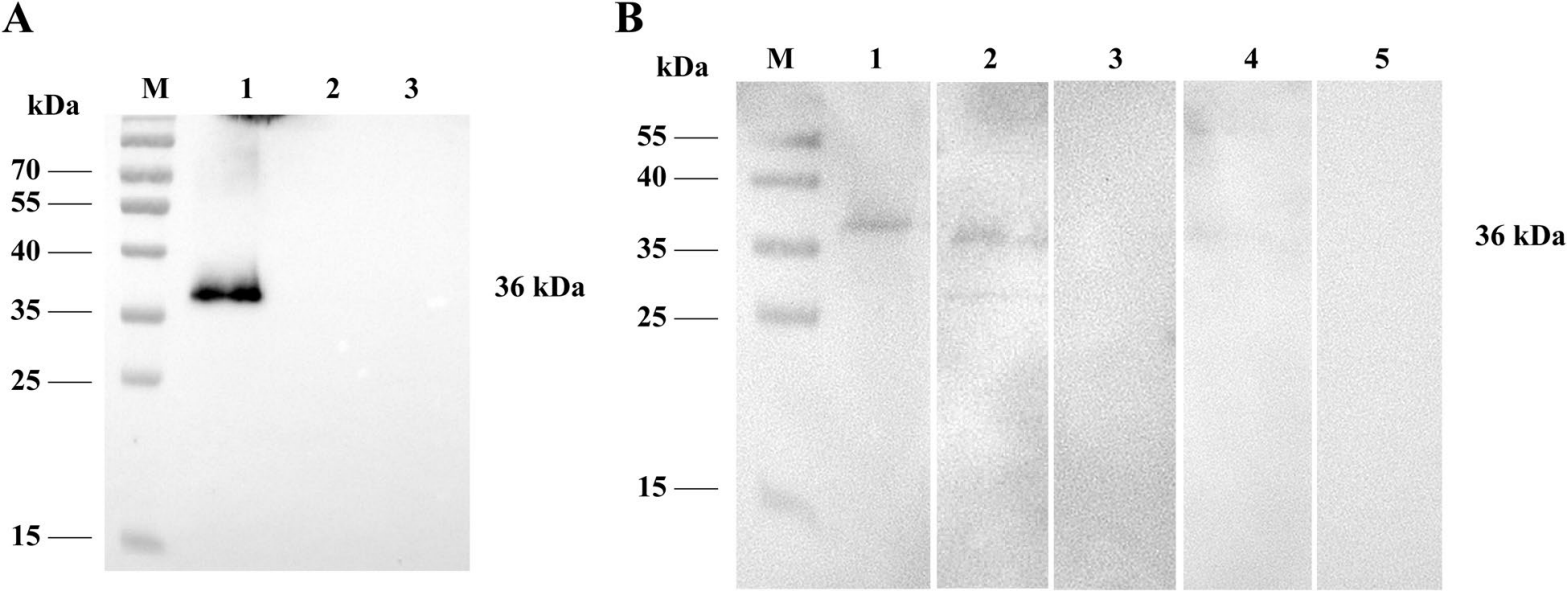
Western blot analysis of rEnMIC3. **A** Identification of recombinant His-EnMIC3 by Western blotting using anti-His monoclonal antibody as the primary antibody and horseradish peroxidase (HRP)-conjugated goat anti-mouse IgG as the secondary antibody. Lane M: protein marker; Lane 1: purified rEnMIC3; Lane 2: pET28a( +)/BL21 with IPTG induction; Lane 3: BL21 with IPTG induction. **B** Western blot analysis of purified rEnMIC3 using convalescent sera from chickens infected with *Eimeria necatrix* (Lane 1), *E. tenella* (Lane 2), *E. acervulina* (Lane 3), and *E. maxima* (Lane 4), with negative control serum in Lane 5. Lane M: protein marker

The cDNA sequence lacking the signal peptide was inserted into the pET28a( +) vector, which contains an N-terminal 6 × His tag. The recombinant expression plasmid pET28a( +)-EnMIC3 was successfully constructed and transformed into *E. coli* BL21(DE3) host cells (Additional file 2: Fig. S4A). Following IPTG induction, the expressed proteins were analyzed by 12% SDS-PAGE and Western blotting. The results showed that the recombinant protein rEnMIC3 migrated at approximately 36 kDa (Additional file 2: Fig. S4B, lane 2), consistent with the predicted molecular weight, and was recognized by anti-His monoclonal antibody (Fig. 3A, lane 1). The protein was predominantly present as inclusion bodies (Additional file 2: Fig. S4B, lane 5) and was subsequently purified using Ni–NTA affinity chromatography to achieve high purity (Additional file 2: Fig. S4C, lane 3).

To evaluate the immunoreactivity of rEnMIC3, Western blot analysis was performed using convalescent sera from chickens infected with different *Eimeria* species. The recombinant protein was specifically recognized by serum from chickens infected with *E. necatrix*, showing a band of approximately 36 kDa (Fig. 3B, lane 1), thereby confirming its antigenic properties. Notably, rEnMIC3 was also recognized by convalescent serum from *E. tenella*-infected chickens (Fig. 3B, lane 2), indicating cross-reactivity between these two closely related species. In contrast, sera from chickens infected with *E. acervulina* or *E. maxima* showed no detectable reactivity with rEnMIC3 (Fig. 3B, lanes 3–4), consistent with the negative control serum from uninfected chickens (Fig. 3B, lane 5).

### Localization and dynamic expression of EnMIC3

**Fig. 4:**
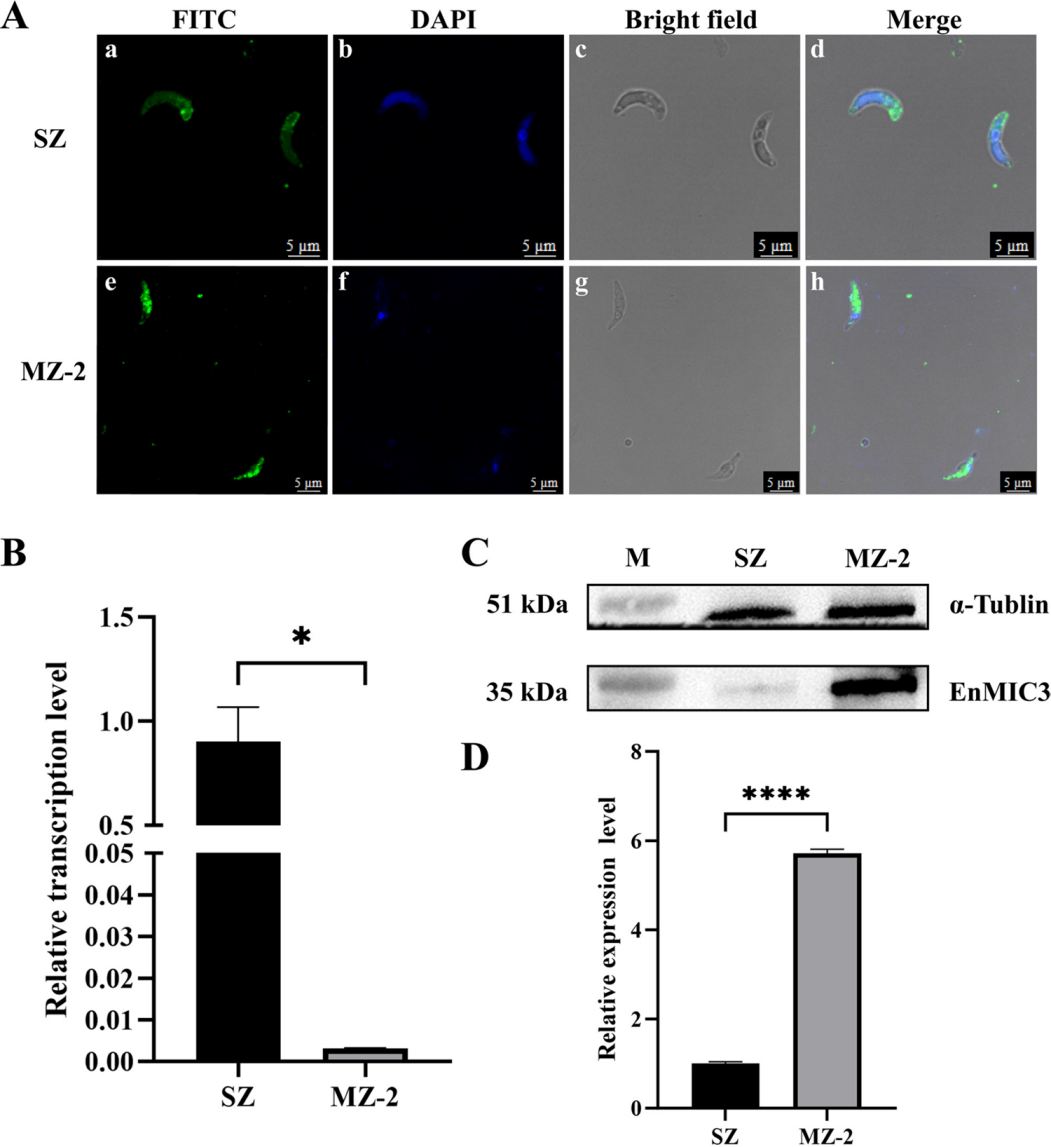
Dynamic expression and localization of EnMIC3. **A** Immunofluorescence localization of EnMIC3 in SZ and MZ-2 using anti-rEnMIC3 polyclonal antibody. a, e: Stained with FITC-conjugated goat anti-mouse IgG; b, f: counterstained with DAPI; c, g: bright-field images; d, h: merged images. Scale bar = 5 µm. **B** Relative transcription levels of En*MIC3* in SZ and MZ-2. *P < 0.05, **P < 0.01, ***P < 0.001, ****P < 0.0001. **C** Western blot analysis of EnMIC3 protein levels in SZ and MZ-2 of *Eimeria necatrix*. **D** Quantitative analysis of EnMIC3 relative protein expression levels based on Western blot. *P < 0.05, **P < 0.01, ***P < 0.001, ****P < 0.0001

IFA revealed that the EnMIC3 protein was specifically localized at the apical end of SZ, whereas it was diffusely distributed throughout the cytoplasm in MZ-2 (Fig. 4A), demonstrating the expression of EnMIC3 in different developmental stages of *E. necatrix*.

*q*RT-PCR analysis showed that the transcription level of En*MIC3* gene was significantly higher in SZ than in MZ-2 (*P* < 0.05) (Fig. 4B). Western blotting analysis detected EnMIC3 protein (35 kDa) in both SZ and MZ-2 of *E. necatrix* (Fig. 4C). Densitometric analysis of the immunoblot suggested a higher EnMIC3 protein level in MZ-2 than in SZ (*P* < 0.0001) (Fig. 4D), although this trend was not consistent with the *q*RT-PCR result.

### EnMIC3's ability to invade host cells

**Fig. 5:**
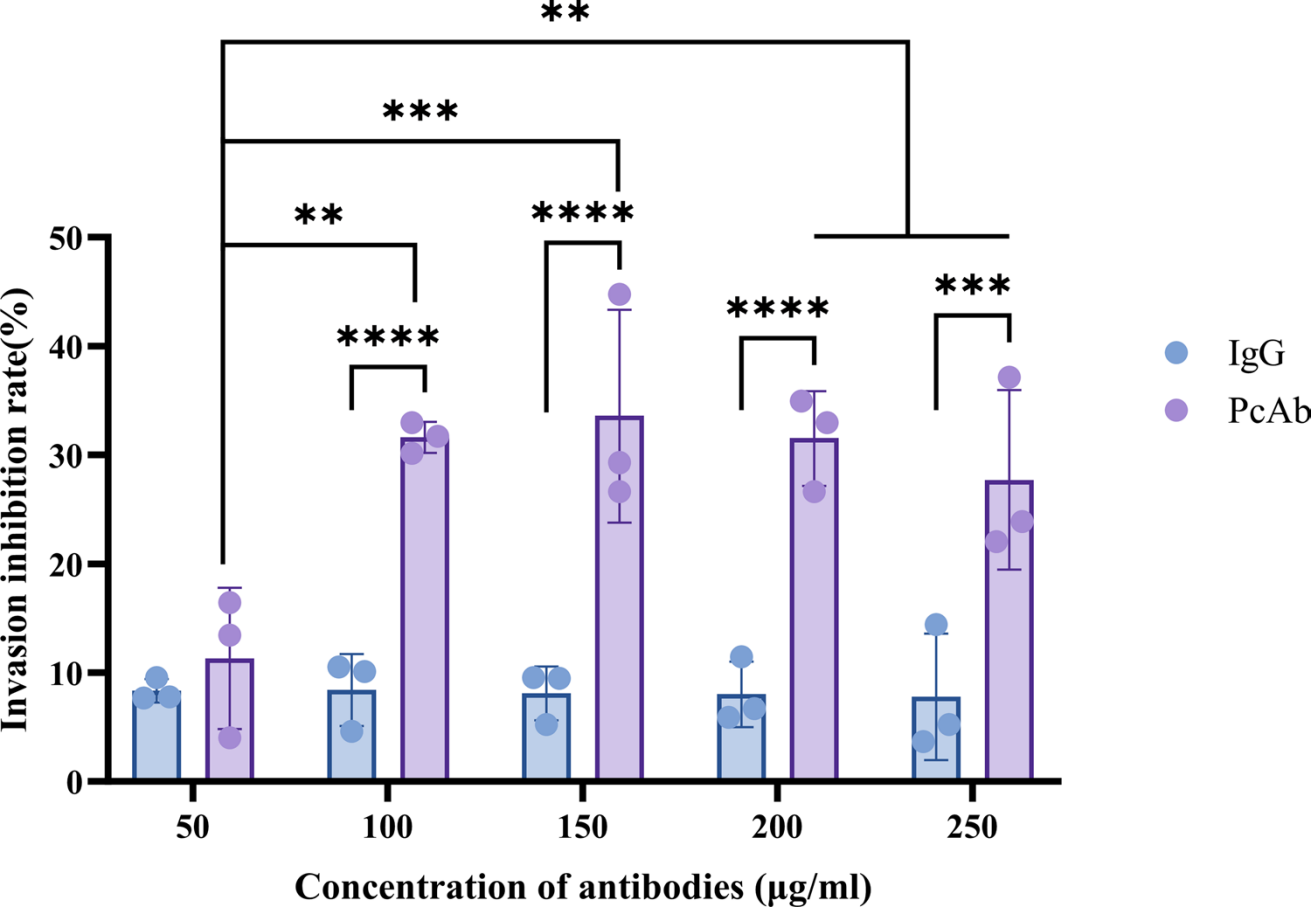
Inhibition of sporozoite invasion by anti-EnMIC3 antibody in vitro. Invasion-inhibitory activities of anti-EnMIC3 antibody at different concentrations. The assays were performed in triplicate. Negative mouse IgG was performed as control. *P < 0.05, **P < 0.01, ***P < 0.001, ****P < 0.0001

To investigate the role of EnMIC3 in sporozoite invasion, polyclonal antibody inhibition assays were performed and analyzed using flow cytometry. At 50 µg/ml, no significant difference in invasion inhibition was observed between the anti-rEnMIC3 group and the mouse IgG control group. However, pretreatment of sporozoites with anti-rEnMIC3 serum at concentrations of 100, 150, 200, and 250 µg/ml significantly inhibited sporozoite invasion of DF-1 cells compared with the mouse IgG control group (*P* < 0.001). The maximum inhibition rate of 33.57% was observed at 150 µg/ml, compared with 8.11% in the mouse IgG control group at the same concentration (Fig. 5). These results demonstrate that antibodies targeting EnMIC3 can partially block sporozoite invasion of host cells.

### Protective efficacy of rEnMIC3

The protective efficacy of rEnMIC3 against *E. necatrix* infection was evaluated using ACI, which incorporates survival rate, body weight gain, intestinal lesion scores, and oocyst reduction. Results for each parameter are presented in Table 3. No mortality was observed in any group following *E. necatrix* challenge. All rEnMIC3-immunized groups demonstrated greater body weight gain than the UC group (*P* < 0.05). Among the immunized groups, the IC-H group achieved the highest BWG (231.10 ± 17.73 g, RBWG 98.82%), which was comparable to the UU group (*P* > 0.05). Intestinal lesion scores and oocyst shedding were substantially reduced in all immunized groups, with the IC-L group showing the lowest lesion score (1.55 ± 0.50) and the highest oocyst reduction rate (71.29%). The ACI values indicated overall protective efficacy, with IC-H achieving the highest ACI of 171.32, followed by IC-L (167.73) and IC-M (161.59), all of which were superior to the UC group (103.87). These results demonstrate that rEnMIC3 confers effective protection against challenge with 2.5 × 10^4^ *E. necatrix* sporulated oocysts, with the IC-H achieving the highest ACI value, primarily due to superior BWG performance.

**Table 3:** Protective effect of the rEnMIC3 against oocysts of *Eimeria necatrix* infection

Group	SR (%)	BWG (g)	RBWG (%)	OP per bird (10^5^)	OR (%)	LS	LI	OI	ACI
IC-H	100	231.10 ± 17.73^a^	98.82	1.45	64.11	1.75 ± 0.35^b^	17.50	10.00	171.32
IC-M	100	205.99 ± 36.18^a^	88.09	1.28	68.32	1.65 ± 0.75^b^	16.50	10.00	161.59
IC-L	100	218.01 ± 16.12^a^	93.23	1.16	71.29	1.55 ± 0.50^b^	15.50	10.00	167.73
UC	100	150.53 ± 14.90^b^	64.37	4.04	0.00	2.05 ± 0.60^b^	20.50	40.00	103.87
UU	100	233.85 ± 20.21^a^	100.00	NA	100.00	0.00 ± 0.00^a^	0.00	0.00	200.00

*IC-H* immunized with a high dose of rEnMIC3 and challenged with oocyst group, *IC-M* immunized with a middle dose of rEnMIC3 and challenged with oocyst group, *IC-L* immunized with a low dose of rEnMIC3 and challenged with oocysts group, *UC* unimmunized and challenged with oocyst group, *UU* unimmunized and unchallenged with oocysts group, *SR* survival rate, *BWG* body weight gain; *RBWG* relative body weight gain, *OP* oocyst production, *OR* oocyst reduction, *LS* lesion score, *LI* lesion index, *OI* oocyst index, *ACI* anticoccidial index. Oocyst output of UU group was zero and shown as “NA.” Different superscript letters (a, b) denote statistically significant differences between groups at *P* < 0.05

### Serum antibody levels against rEnMIC3

**Fig. 6:**
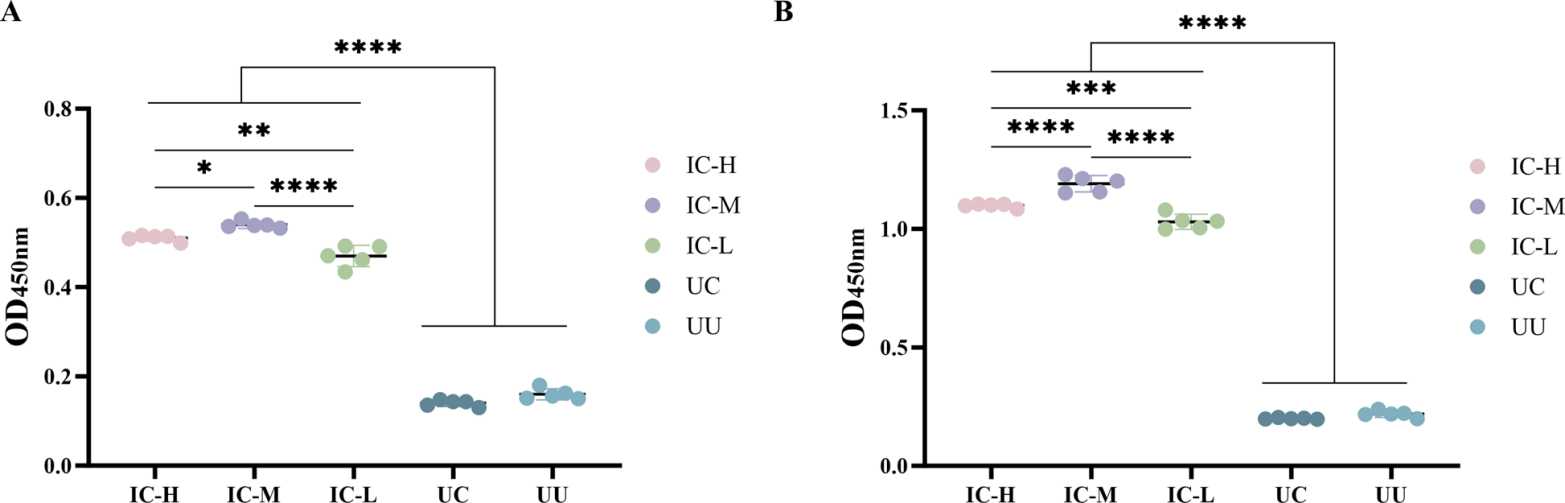
The antibody levels in the serum after immunization. **A** After the first immunization and after the second immunization (**B**). *P < 0.05, **P < 0.01, ***P < 0.001, ****P < 0.0001

To evaluate the humoral immune responses induced by rEnMIC3 immunization, serum antibody levels were measured. Following primary immunization, a significant increase in antibody levels was observed in all rEnMIC3-immunized groups compared with control groups on day 14 (*P* < 0.0001), with the IC-M group exhibiting the highest serum antibody levels among the five groups (Fig. 6A). After the booster immunization, antibody levels were substantially further increased in all immunized groups on day 21. Notably, the IC-M group achieved the highest antibody titer, which was significantly higher than those of both the IC-H (*P* < 0.0001) and the IC-L groups (*P* < 0.05) (Fig. 6B).

## Discussion

Micronemes are conserved and specialized apical secretory organelles found in apicomplexan parasites. Upon contact with host cells, MICs are redistributed to the parasite surface and participate in parasite-host cell interactions [12, 32]. Many MICs contain characteristic adhesive domains, including thrombospondin type I (TSP-1), epidermal growth factor (EGF), and Apple domains [12, 33]. In *Eimeria* species, at least 11 MICs have been reported to date, including MIC1-8 and apical membrane antigens (AMA) 1–3 [11, 19]. Among these, MIC3 has been a particular focus of investigation due to its critical role in host cell invasion and has been characterized in *E. tenella*, *E. acervulina*, *E. maxima*, and *E. mitis* [19, 21]. In the present study, we cloned and characterized EnMIC3, a protein that encodes a 265-amino acid protein containing an N-terminal signal peptide and an EGF-like domain. These features align with those observed in known MICs, confirming that EnMIC3 represents a microneme protein in *E. necatrix*.

The EnMIC3 sequence from the Yangzhou strain lacks a tandem repeat of four threonine residues compared with the Houghton strain (GenBank accession no. XP_013432984.1). Importantly, this deletion does not alter the predicted antigenic epitopes or conserved functional domains. Therefore, it may represent a naturally occurring sequence variation between isolates; however, additional analyses involving more geographically diverse isolates are required to confirm its distribution and potential functional significance. Western blot analysis showed that the native EnMIC3 protein migrates at approximately 35 kDa, which is notably higher than the predicted 28.5 kDa. This anomalous mobility is consistent with post-translational glycosylation, a common modification observed in apicomplexan MICs [34]. Phylogenetic analyses revealed that EnMIC3 clusters most closely with *E. tenella* MIC3 (EtMIC3), which may explain the previously observed serological cross-reactivity between different isolates. Notably, the phylogenetic clustering of MIC3 homologs appears to align with species-specific intestinal localization, supporting the hypothesis that MIC3 has undergone divergent evolution as it adapts to distinct intestinal ecological niches among *Eimeria* species [19]. The phylogenetic tree also revealed a clear separation between short-form MIC3 proteins (including EnMIC3) and long-form MIC3 proteins from *E. tenella*, *E. mitis*, *E. maxima*, and *E. acervulina*. The long-form MIC3 proteins harbor significantly more MAR domains, which function as sialic acid-binding lectins mediating host cell recognition [35, 36], and specific MAR domains have been shown to determine the site specificity of parasite infection along the chicken intestinal tract [19, 37]. In contrast, EnMIC3 has a simpler domain architecture with a single EGF-like domain, raising the question of how this short-form MIC3 contributes to invasion.

Adhesion and invasion are critical steps in host cell infection by apicomplexan parasites. MICs play essential roles in these processes through their adhesive domains [33]. EGF-like domains are evolutionarily conserved motifs found in apicomplexan MICs, characterized by six cysteine residues that form three intramolecular disulfide bonds [38, 39]. These domains facilitate protein folding, mediate complex formation between MICs, and enable microneme targeting through interactions with transmembrane escorter proteins [40]. In addition, EGF-like domains may participate directly in receptor recognition or indirectly enhance parasite attachment by maintaining the proper spatial organization of adhesive regions[33]. Sequence analysis revealed that EnMIC3 contains a single EGF-like domain. Although the number of EGF domains in EnMIC3 is less than that observed in some previously reported MIC proteins—such as EtMIC8, which has five EGF-like domains [11], or TgMIC6, which has three EGF-like domains [41]—a single EGF domain may still play a critical role in protein folding, complex formation, or receptor recognition.

Antibody blocking assays demonstrated that anti-rEnMIC3 polyclonal antibodies inhibited sporozoite invasion of DF-1 cells in a dose-dependent manner, achieving a maximum inhibition rate of 33.57% at 150 µg/ml, significantly higher than the control IgG group (~ 10%). These findings support the functional involvement of EnMIC3 in host cell invasion, consistent with previous studies [11, 42]. The relatively moderate inhibition rate may be attributed to the presence of only a single EGF domain, as MICs containing multiple tandem EGF repeats typically exhibit stronger adhesive capacity through synergistic enhancement of receptor binding [33]. Moreover, the inhibitory effect of anti-rEnMIC3 antibodies plateaued at concentrations > 150 µg/ml. This phenomenon may result from saturation of accessible epitopes on the sporozoite surface. Alternatively, this plateau effect may reflect the involvement of redundant invasion pathways mediated by other adhesins. These findings suggest that while EnMIC3 participates in host cell invasion, it may not serve as the predominant adhesin in this process but rather functions in concert with other MICs. However, these interpretations remain speculative and require further experimental validation. Further studies are required to elucidate the precise role of EnMIC3 in parasite invasion. In particular, characterization of its host receptors, interacting partners, and the function of its EGF-like domain will provide deeper insight into the underlying mechanism.

Immunofluorescence analysis revealed that EnMIC3 protein is specifically localized to the apical region in SZ, while exhibiting a cytoplasmic distribution in MZ-2. This pattern is consistent with other coccidian MICs; for instance, EtMIC2 predominantly localizes to the anterior region of sporozoites but shows cytoplasmic distribution in merozoites [43], and EmiMIC3 displays a similar localization shift [18]. The apical localization of EnMIC3 in sporozoites strongly suggests its involvement in initial host cell recognition and attachment processes.

Intriguingly, our results suggested that the mRNA and protein expression patterns of EnMIC3 were not fully consistent across developmental stages: transcript levels were significantly higher in SZ, whereas densitometric analysis suggested that EnMIC3 protein levels were higher in MZ-2. Similar discrepancies between transcript and protein abundance have been reported in apicomplexan parasites. For example, global analyses in *Plasmodium falciparum* reveal temporal lags between peak mRNA levels and protein accumulation [44, 45]. Such post-transcriptional regulation may enable rapid adaptation to environmental fluctuations, potentially explaining its prevalence across Apicomplexa [44]. Additionally, merozoites are enriched in invasion-related proteins, likely reflecting the extensive replication occurring during schizogony [46–48]. Collectively, these findings suggest that EnMIC3 may execute distinct biological functions at different developmental stages—serving as an adhesin critical for host cell recognition and invasion in sporozoites, while potentially fulfilling alternative roles related to intracellular survival or subsequent invasion cycles in merozoites.

Proteins containing EGF domains have emerged as compelling vaccine candidates against apicomplexan parasites. In *Plasmodium* species, recombinant proteins containing the EGF-like domains of merozoite surface proteins (MSP-1, MSP-4/5, MSP-8) confer significant protective immunity against blood-stage infection, with MSP-1 inducing complete protection in murine models [49–51]. Similarly, the EGF-like domains of ookinete surface antigens Pfs25 and Pvs25 elicit potent transmission-blocking antibody responses [52, 53]. In *Toxoplasma gondii*, EGF-containing MICs, including MIC3, MIC6, and MIC8, have demonstrated considerable immunogenic potential, eliciting both humoral and cellular immunity [54–57]. In *Eimeria*, EtMIC4 of *E. tenella* is a remarkable example, containing 31 tandem EGF modules and 12 thrombospondin type-1 (TSP-1) repeats [58]. More recently, immunization with the recombinant EtMIC8-EGF peptide induced significant increases in serum IgG antibody levels and enhanced proportions of CD3⁺CD4⁺ and CD3⁺CD8⁺ T lymphocytes [11]. Consistent with these findings, our results demonstrated that immunization with 200 µg rEnMIC3 elicited superior immunogenicity and protective efficacy, achieving an ACI value of 171.32. Similar protective potential has also been reported for other microneme antigens, including EtMIC2 and EtMIC3, which were shown to enhance immune responses and confer partial protection against *Eimeria* challenge in chickens [43, 59, 60]. Consistently, rEnMIC3 also provided measurable but incomplete protection. This pattern is consistent with the general characteristics of subunit vaccines against coccidiosis, which typically reduce parasite burden and intestinal lesions rather than complete parasite clearance.

In the present study, rEnMIC3 immunization markedly increased antibody levels, indicating effective humoral activation and immunological memory formation. Notably, the superior performance observed at the 200-µg dose suggests that an appropriate antigen dosage is critical for inducing optimal protective immunity, whereas lower or higher doses may result in suboptimal immune responses. Notably, the protective effects were not fully consistent across all evaluation parameters. The low-dose group showed relatively lower oocyst output and lesion scores, but this was not mirrored by body weight gain. Such discrepancies are not uncommon in coccidiosis studies, because these parameters reflect different aspects of infection [61–63]. Moreover, although no mortality was observed in the unvaccinated/challenged group, clear pathological and parasitological changes were still detected, indicating that the challenge model was suitable for evaluating vaccine efficacy under controlled experimental conditions. Collectively, these data further reinforce the potential of EGF-containing microneme proteins as promising vaccine candidates against coccidiosis. Nevertheless, multi-species challenges would be required to assess whether rEnMIC3 provides cross-protection against other *Eimeria* species, which is essential for practical vaccine application, given that chickens under field conditions are frequently exposed to mixed *Eimeria* infections.

## Conclusions

In this study, we identified and characterized EnMIC3 as a functional microneme protein in *E. necatrix*. EnMIC3 localizes to the apical region in sporozoites and participates in host cell invasion. Immunization with rEnMIC3 induced significant antibody responses and provided effective protection against *E. necatrix* infection (ACI: 171.32). These results suggest that EnMIC3 is a promising vaccine candidate for the control of *E. necatrix* coccidiosis in poultry and also lay a foundation for further exploration of the role of MICs in the host invasion and growth development of the parasite.

## Supplementary Information

Additional file 1: Fig. S1. SDS-PAGE analysis of purified mouse anti-rEnMIC3 polyclonal antibody. M: protein ladder; Lane 1: purified mouse polyclonal antibody against rEnMIC3. The heavy chain (~50 kDa) and light chain (~25 kDa) of IgG are indicated. Fig. S2. Percent identity and divergence of the EnMIC3 gene between the Yangzhou strain (EnMIC3) and the Houghton strain (XM_013577530.1). Fig. S3. Multiple sequence alignment of MIC3 proteins from different *Eimeria* species infecting chickens. Conserved, partially conserved, and gap positions are shown in black, gray, and dashes, respectively. The “Majority” line indicates the consensus sequence. The matrix shows pairwise sequence identity (above diagonal) and divergence (below diagonal) for EnMIC3, EtMIC3, EaMIC3, and EbMIC3.

Additional file 2: Fig. S4. Expression and purification of EnMIC3. (A) Restriction enzyme digestion analysis of the recombinant plasmid pET28a(+)-EnMIC3. M: DL5000 DNA Marker; Lane 1: pET28a(+)-EnMIC3 digested with EcoR I and Xho I. (B) Expression and solubility analysis of rEnMIC3. Lane M: protein marker; Lane 1: pET28a(+)-EnMIC3/BL21 without IPTG induction; Lane 2: pET28a(+)-EnMIC3/BL21 with IPTG induction; Lane 3: pET28a(+)/BL21 with IPTG induction; Lane 4: supernatant of pET28a(+)-EnMIC3/BL21 with IPTG induction; Lane 5: pellet of pET28a(+)-EnMIC3/BL21 with IPTG induction. (C) Purification of rEnMIC3. Lane M: protein marker; Lane 1: urea-solubilized inclusion bodies; Lane 2: eluted protein from Ni-NTA; Lane 3: purified recombinant protein after renaturation.

## Data Availability

All data generated or analyzed during this study are included in this published article.

## Abbreviations

ACI: Anticoccidial index
BSA: Bovine serum albumin
DTT: Dithiothreitol
*E. coli*: *Escherichia coli*
EGF: Epidermal growth factor
HRP: Horseradish peroxidase
IFA: Indirect immunofluorescence analyses
IPTG: Isopropyl-β-D-thiogalactopyranoside
ITS: Internal transcribed spacer
MICs: Microneme proteins
MZ-2: Second-generation merozoites
PBS: Phosphate-buffered saline
pI: Isoelectric point
P-PBS: Percoll in PBS
RIPA: Radioimmunoprecipitation assay
SDS–PAGE: Sodium dodecyl sulfate-polyacrylamide gel electrophoresis
SZ: Sporozoites
