# Supplementary figures and images for: Molecular characterization and immunoprotective potential of microneme protein 3 from Eimeria necatrix
Source: Parasit Vectors. 2026 May 2;19:253. doi: 10.1186/s13071-026-07418-w (PMC13281574; doi:10.1186/s13071-026-07418-w)

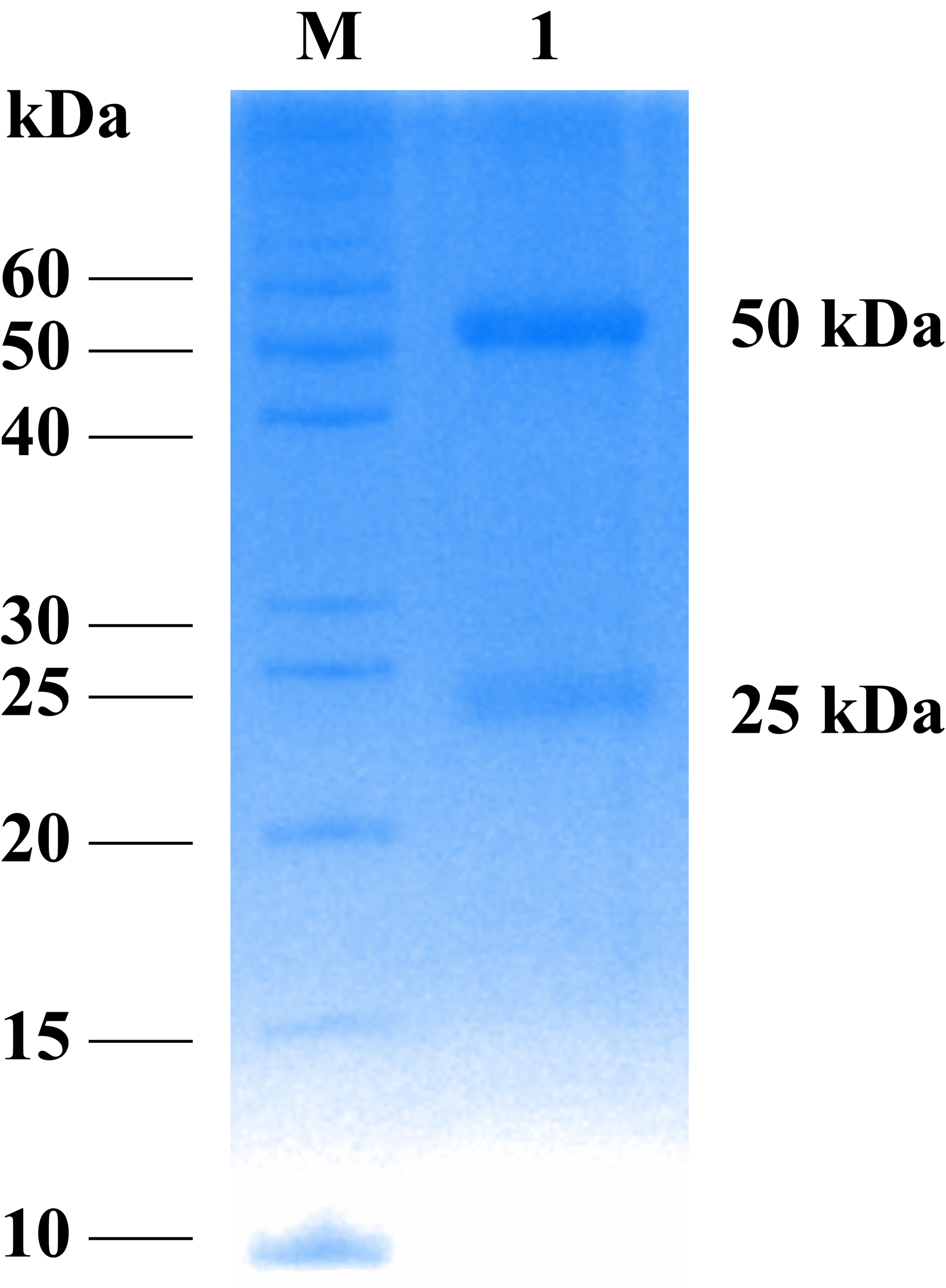

Supplement: Supplementary file 1 — Additional file 1: Fig. S1. SDS-PAGE analysis of purified mouse anti-rEnMIC3 polyclonal antibody. M: protein ladder; Lane 1: purified mouse polyclonal antibody against rEnMIC3. The heavy chain (~50 kDa) and light chain (~25 kDa) of IgG are indicated. Fig. S2. Percent identity and divergence of the EnMIC3 gene between the Yangzhou strain (EnMIC3) and the Houghton strain (XM_013577530.1). Fig. S3. Multiple sequence alignment of MIC3 proteins from different Eimeria species infecting chickens. Conserved, partially conserved, and gap positions are shown in black, gray, and dashes, respectively. The “Majority” line indicates the consensus sequence. The matrix shows pairwise sequence identity (above diagonal) and divergence (below diagonal) for EnMIC3, EtMIC3, EaMIC3, and EbMIC3. [file 13071_2026_7418_MOESM1_ESM.zip › Additional file 1/Figure S1.tif]

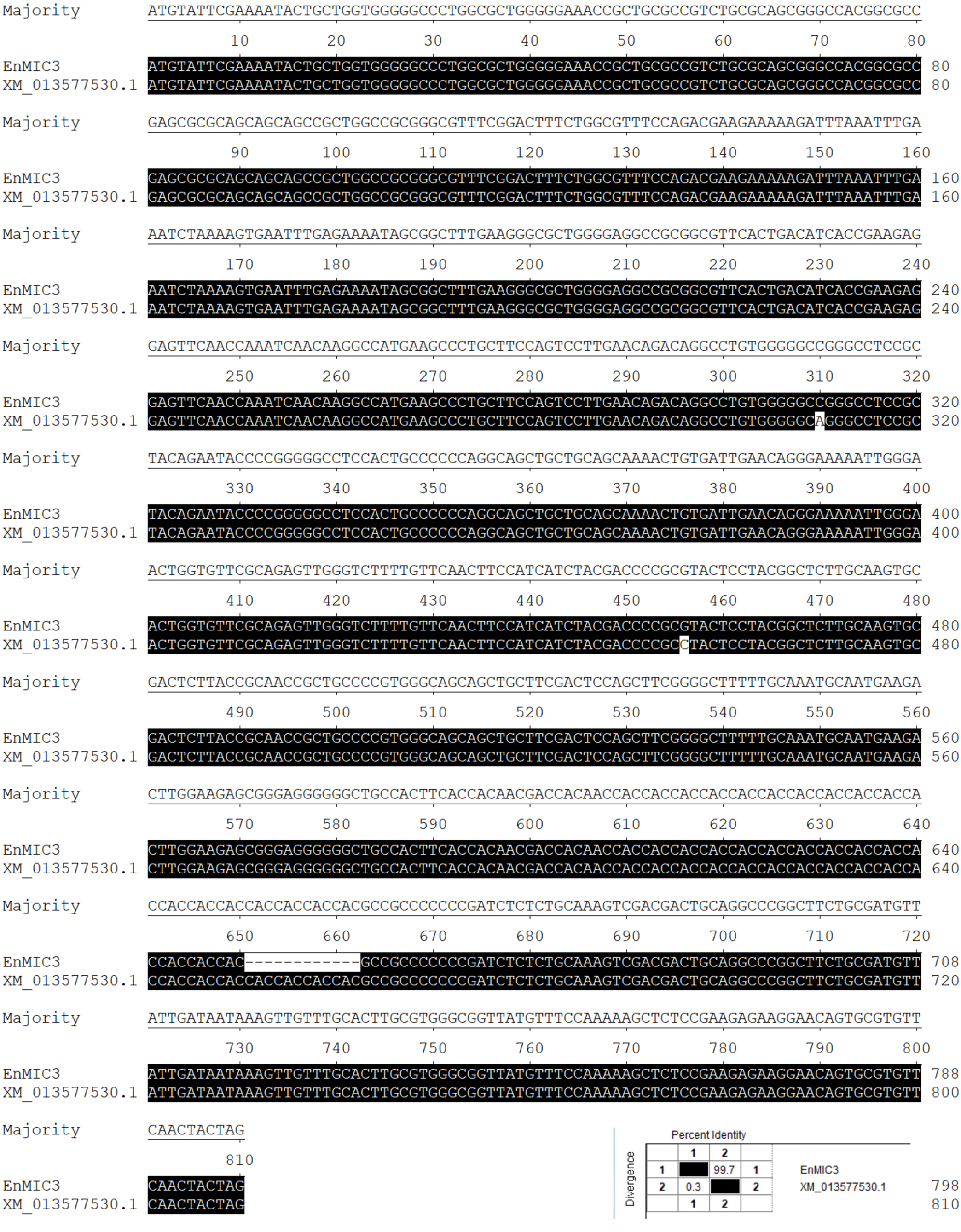

Supplement: Supplementary file 1 — Additional file 1: Fig. S1. SDS-PAGE analysis of purified mouse anti-rEnMIC3 polyclonal antibody. M: protein ladder; Lane 1: purified mouse polyclonal antibody against rEnMIC3. The heavy chain (~50 kDa) and light chain (~25 kDa) of IgG are indicated. Fig. S2. Percent identity and divergence of the EnMIC3 gene between the Yangzhou strain (EnMIC3) and the Houghton strain (XM_013577530.1). Fig. S3. Multiple sequence alignment of MIC3 proteins from different Eimeria species infecting chickens. Conserved, partially conserved, and gap positions are shown in black, gray, and dashes, respectively. The “Majority” line indicates the consensus sequence. The matrix shows pairwise sequence identity (above diagonal) and divergence (below diagonal) for EnMIC3, EtMIC3, EaMIC3, and EbMIC3. [file 13071_2026_7418_MOESM1_ESM.zip › Additional file 1/Figure S2.tif]

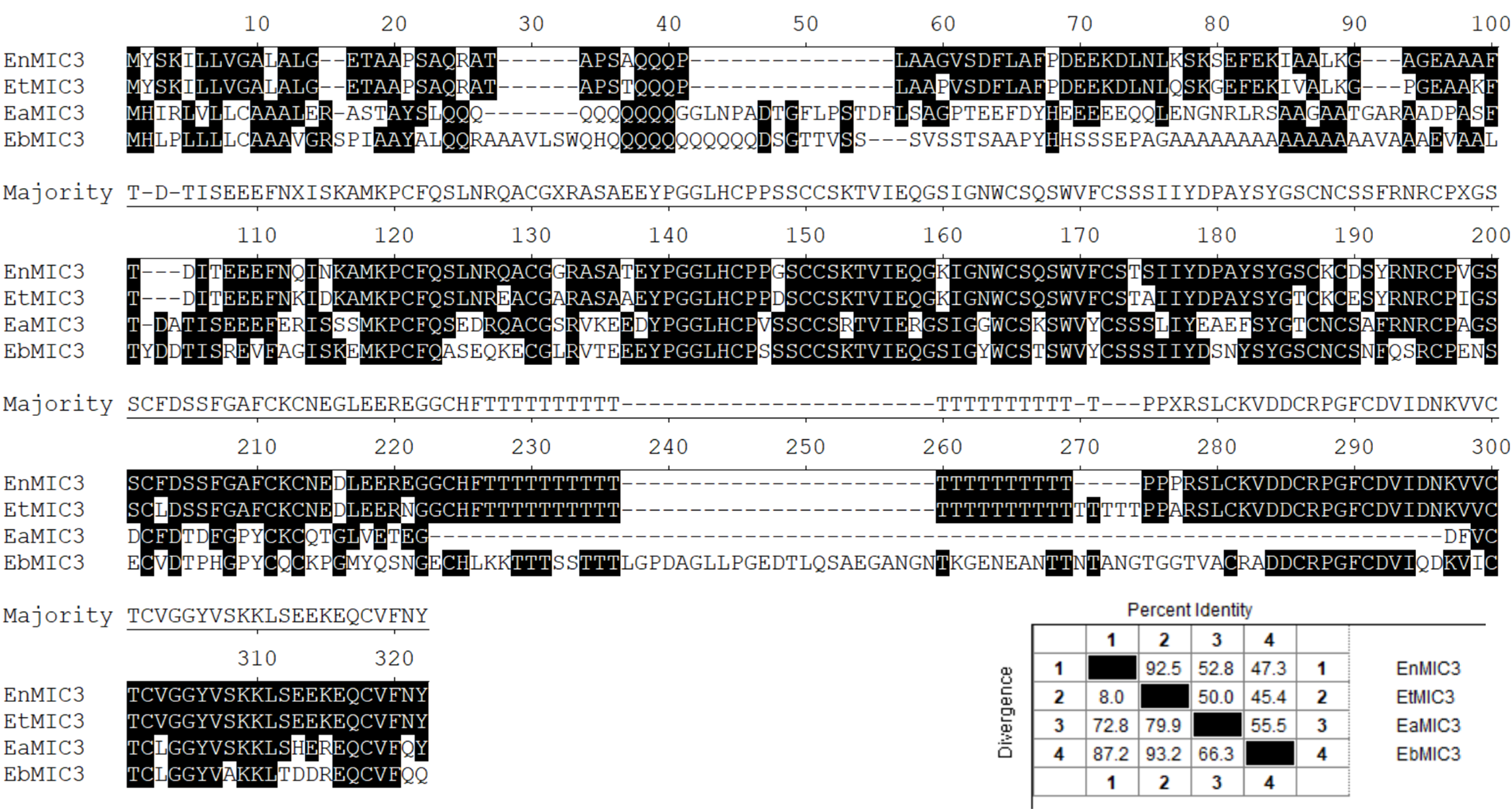

Supplement: Supplementary file 1 — Additional file 1: Fig. S1. SDS-PAGE analysis of purified mouse anti-rEnMIC3 polyclonal antibody. M: protein ladder; Lane 1: purified mouse polyclonal antibody against rEnMIC3. The heavy chain (~50 kDa) and light chain (~25 kDa) of IgG are indicated. Fig. S2. Percent identity and divergence of the EnMIC3 gene between the Yangzhou strain (EnMIC3) and the Houghton strain (XM_013577530.1). Fig. S3. Multiple sequence alignment of MIC3 proteins from different Eimeria species infecting chickens. Conserved, partially conserved, and gap positions are shown in black, gray, and dashes, respectively. The “Majority” line indicates the consensus sequence. The matrix shows pairwise sequence identity (above diagonal) and divergence (below diagonal) for EnMIC3, EtMIC3, EaMIC3, and EbMIC3. [file 13071_2026_7418_MOESM1_ESM.zip › Additional file 1/Figure S3.tif]
